# Impacts of Priming with Silicon on the Growth and Tolerance of Maize Plants to Alkaline Stress

**DOI:** 10.3389/fpls.2016.00243

**Published:** 2016-03-10

**Authors:** Arafat A. Abdel Latef, Lam-Son P. Tran

**Affiliations:** ^1^Botany Department, Faculty of Science, South Valley UniversityQena, Egypt; ^2^Biology Department, College of Applied Medical Science, Taif UniversityTaif, Saudi Arabia; ^3^Plant Abiotic Stress Research Group & Faculty of Applied Sciences, Ton Duc Thang UniversityHo Chi Minh City, Vietnam; ^4^Signaling Pathway Research Unit, RIKEN Center for Sustainable Resource ScienceYokohama, Japan

**Keywords:** alkaline stress, antioxidant enzymes, leaf pigments, maize, osmoprotectants, seed-priming with Si, stress mitigation

## Abstract

Silicon (Si) has been known to augment plant defense against biotic and abiotic pressures. Maize (*Zea maize* L.) is classified as a Si accumulator and is relatively susceptible to alkaline stress. In this study, seeds of maize were grown in pots and exposed to various concentrations of Na_2_CO_3_ (0, 25, 50, and 75 mM) with or without 1.5 mM Si in the form of sodium metasilicate Na_2_O_3_Si.5H_2_O for 25 days. Alkaline-stressed plants showed a decrease in growth parameters, leaf relative water content (LRWC), and the contents of photosynthetic pigments, soluble sugars, total phenols and potassium ion (K^+^), as well as potassium/sodium ion (K^+^/Na^+^) ratio. By contrast, alkaline stress increased the contents of soluble proteins, total free amino acids, proline, Na^+^ and malondialdehyde (MDA), as well as the activities of superoxide dismutase (SOD), catalase (CAT), and peroxidase (POD) in stressed plants. On the other hand, application of Si by seed-priming improved growth of stressed plants, which was accompanied by the enhancement in LRWC, and levels of photosynthetic pigments, soluble sugars, soluble proteins, total free amino acids and K^+^, as well as activities of SOD, CAT, and POD enzymes. Furthermore, Si supplement resulted in a decrease in the contents of proline, MDA and Na^+^, which together with enhanced K^+^ level led to a favorable adjustment of K^+^/Na^+^ ratio, in stressed plants relative to plants treated with alkaline stress alone. Taken together, these results indicate that Si plays a pivotal role in alleviating the negative effects of alkaline stress on maize growth by improving water status, enhancing photosynthetic pigments, accumulating osmoprotectants rather than proline, activating the antioxidant machinery, and maintaining the balance of K^+^/Na^+^ ratio. Thus, our findings demonstrate that seed-priming with Si is an efficient strategy that can be used to boost tolerance of maize plants to alkaline stress.

## Introduction

Alkaline stress, which is defined as the existence of alkaline salts (Na_2_CO_3_ or NaHCO_3_) in the soil (Paz et al., 2012), is one of the most crucial abiotic stressors which plants encounter in the era of climate change. A number of studies have shown that alkaline stress is more dangerous than saline stress, owing to its additional high pH stress (Campbell and Nishio, 2000; Hartung et al., 2002; Chen et al., 2012; Radi et al., 2012). High pH value may lead to reduction in seed germination, destruction of the root cell structure, change in the nutrient availability and disorder in nutrient uptake, and thus resulting in a significant decrease in the yield of agricultural plants (Peng et al., 2008; Gao et al., 2014). A few studies have been carried out on the effects of alkaline stress on plant growth and productivity. However, only scant information is available about the morphological, physiological, biochemical, and antioxidative responses in plants under alkaline stress. Egyptian soils are in general distinguished by a little alkaline to alkaline pH values (7.5–8.7) which are mainly due to its arid ambience (Abd-Alla et al., 2014).

Priming of seeds with silicon (Si) is one of the major techniques, which can improve abiotic stress tolerance in plants (Hameed et al., 2013). Si is the second most abundant element found in soil, next to oxygen, and the eighth most common element in nature (Sahebi et al., 2015). It is recognized as ‘quasi essential’ element for plants because its deficiency results in various dysfunctions with respect to plant growth, evolution, and proliferation (Hasanuzzaman et al., 2013). Si, as a fertilizer, biostimulant or plant protectant, plays a pivotal role in enhancing the plants growth and productivity, especially in stress regimes (Sawas and Ntatsi, 2015). Over the last two decades, numerous laboratory, greenhouse and field experiments have demonstrated that Si is able to hamper both biotic pressures caused by plant diseases and pest attacks, as well as abiotic pressures, including physical pressures such as drought, waterlogging, freezing, high temperature, and UV, and chemical pressures as salinity, nutrient deficiencies, and metal toxicity (for reviews, see Guntzer et al., 2012; Balakhnina and Borkowska, 2013; Marafon and Endres, 2013; Van Bockhaven et al., 2013; Zhu and Gong, 2014; Rizwan et al., 2015).

Maize (Zea *mays* L.) is the third most cultivated cereal crop in total world production after wheat and rice (Malčovská et al., 2014a). Maize is one of the important crops in Egypt due to its significance as a feed crop, being used in a number of foods and in oil, starch and pharmaceutical industries, as well as newly emerging as a biofuel crop (Ali et al., 2015). Unluckily, in many regions, especially in the tropics and sub-tropics, the productivity of maize is markedly reduced due to alkalinity. Maize has been known as a Si accumulator (Liang et al., 2006); and thus it is a popular crop for studies on the useful impacts of Si under environmental pressures (Malčovská, 2014b).

Despite application of Si has been investigated in different plants grown under various abiotic stresses, literature has not yet been available regarding the strategies of the Si-mediated protective effects under alkaline stress. Thus, in the present study, we attempted to explain the impacts of Si on growth and tolerance of maize plants subjected to alkaline stress. Therefore, we designed a series of experiments to examine the possible promotion effects of Si pre-treatment on growth traits, the contents of photosynthetic pigments, osmoprotectants, total phenols, sodium ion (Na^+^), potassium ion (K^+^), and malondialdehyde (MDA), as well as the activities of antioxidant enzymes of maize seedlings grown in soil irrigated with different concentrations of alkaline salt.

## Materials and Methods

### Plant Growth and Treatments

This experiment was conducted in the wire-house of the experimental farm of South Valley University, Qena, Egypt located at latitude 26°11′ 25′′ N and longitude 32° 44′ 42′′ E. Plants were grown under natural conditions of temperature, light, and humidity during growing season 2015 (from May 15^th^ to June 8^th^). Seeds of maize (*Zea maize* L. cv. single hybrid 10) were surface-sterilized with mercuric chloride (0.1%) for 5 min, and then rinsed three times with distilled water. The sterilized seeds were divided into two groups; the first group was primed with distilled water, and the second group with 1.5 mM of freshly prepared Si (as sodium metasilicate Na_2_O_3_Si.5H_2_O) solution for 12 h, thereafter air-dried. The seeds of both groups were sown in plastic pots (five seeds/pot) filled with 2 kg of dried soil. The pots were arranged in completely randomized design in factorial arrangement with three replications. At the time of sowing, the seeds were irrigated at field capacity with various alkaline salt concentrations of 0 (control), 25, 50, and 75 mM Na_2_CO_3_ based on the method of Radi et al. (2012); with each pot receiving 400 ml of a designated saline solution. The Na_2_CO_3_ concentrations used were equivalent to 0 (control), 0.528, 1.056, and 1.584 g Na_2_CO_3_ kg^-1^ soil, respectively. Leaching was avoided by maintaining soil water below field capacity at all times. The Si and Na_2_CO_3_ concentrations were selected based on our preliminary tests. The pots were then irrigated at field capacity with normal water through the whole experimental period (25 days). After 25 days after sowing the maize plants were harvested for further analyses.

### Growth Criteria

The fresh weight of maize seedlings was estimated after harvesting. The harvested seedlings were washed with deionized water, and blotted on paper towels before being weighed. Their dry weight was estimated after oven drying at 80°C to constant weight. The dried tissues were powderized and stored in clean sealed glasses at room temperature for later analysis. Leaf relative water content (LRWC) was determined using the method described in Garíca-Mata and Lamattina (2001), using the equation: RWC (%) = (Fresh weight – Dry weight/Turgid weight – Dry weight) × 100. Leaf area was measured using a Planimeter (SOKKIA PLANIMELER KP-90 UK).

### Photosynthetic Pigments

The contents of chlorophyll (Chl) a and b, and carotenoids in fresh leaves were assessed spectrophotometrically according to Lichtenthaler and Wellburn (1983). The 0.05 g of fully expanded young leaves of 25-day-old plants were used for pigment extraction in 80% acetone. The extract of pigments was measured versus a blank of pure 80% acetone at 663, 644, and 452.5 nm for Chl a, Chl b, and carotenoid contents, respectively.

### Estimation of Osmoprotectant Contents

Total soluble sugar content in 25-day-old plants was determined by the anthrone sulfuric acid method according to Irigoyen et al. (1992). Total soluble protein content in 25-day-old plants was determined according to Bradford (1976). The contents of total free amino acids of 25-day-old plants tissues were determined according to the method of Lee and Takanashi (1966). The content of proline in 25-day-old plants was measured according to the method described by Bates et al. (1973).

### Estimation of Total Phenol Content

Total phenol content in 25-day-old plants was assayed by Folin–Ciocalteu reagent (Skerget et al., 2005). The 2.5 mL of Folin–Ciocalteu reagent (diluted 10 times with water) and 2 mL of sodium carbonate (7.5% w/v) solution were added to 0.5 mL of sample extract (0.25 mg mL^-1^) in water. After 20 min of incubation at room temperature, the absorbance was measured spectrophotometrically at 760 nm. Total phenols were quantified by calibration curve obtained from measuring the known concentrations of standard gallic acid (0–100 μg mL^-1^). The phenolic contents of the samples were expressed as mg of GAE (gallic acid equivalent) g^-1^ dry weight of sample.

### Estimation of Na^+^ and K^+^ Levels

Dried powder samples (0.1 g) were treated with perchloric acid 80% (2 mL) and concentrated H_2_SO_4_ (10 mL) for 12 h. Each digested material was then diluted with distilled water to a definite volume (100 mL). The contents of Na^+^ and K^+^ were determined by flame photometry according to the method described by Williams and Twine (1960).

### Determination of MDA Content

Malondialdehyde content was determined according to the thiobarbituric acid (TBA) reaction as described by Zhang and Qu (2004). Fresh leaf sample (0.5 g) was homogenized with 5% trichloroacetic acid and centrifuged at 4,000 × *g* for 10 min. Two milliliter of extract was mixed with 2 mL of 0.6% TBA, and the mixture was placed in a boiling water bath for 10 min. Subsequently, the absorbances were read at 532, 600, and 450 nm. The MDA content was calculated using the formula: 6.45 × (*A*_532_ - *A*_600_) - 0.56 × *A*_450_.

### Assays for Antioxidant Enzyme Activities

#### Extraction of Enzymes

Samples were extracted from the fresh leaves based on the method of Mukherjee and Choudhari (1983). The fresh leaves (0.5 g) were frozen in liquid nitrogen and ground in 10 mL of 100 mM phosphate buffer (KH_2_PO_4_/K_2_HPO_4_) pH 7.0, containing 0.1 mM Na_2_EDTA and 0.1 g of polyvinylpyrrolidone (PVP). The homogenate was centrifuged at 15,000 × *g* at 4°C for 10 min. Subsequently, the supernatant was stored at 4°C until being used for superoxide dismutase (SOD), catalase (CAT), and peroxidase (POD) assays.

#### Assay for SOD Activity

The activity of SOD (EC 1.15.1.1) was determined using the nitro blue tetrazolium (NBT) method described by Giannopolitis and Ries (1977). One unit of SOD was defined as the amount of enzyme required to cause 50% inhibition of the reduction of NBT as monitored at 560 nm.

#### Assay for CAT Activity

Catalase (EC 1.11.1.6) activity was measured according to the method previously described (Aebi, 1984). The CAT activity was assayed by the decline of absorbance at 240 nm as a consequence of H_2_O_2_ consumption.

#### Assay for POD Activity

Peroxidase (EC 1.11.1.7) activity was determined using the method of Maehly and Chance (1954) by assessing the oxidation rate of guaiacol in the presence of H_2_O_2_ at 470 nm (Klapheck et al., 1990).

### Statistical Analysis

All data shown are the mean values. Data were statistically analyzed by the analysis of variance (ANOVA) with SAS software (Version 9.1; SAS Institute, Cary, NC, USA) using Duncan’s multiple range test at the 0.05 level of significance (*p* < 0.05). Data represented in the Tables and Figures are means ± standard deviation (SD) of three independent replicates of each treatment.

## Results

### Positive Impacts of Si on Maize Growth Parameters

Data shown in **Table 1** indicated that the values of fresh and dry weights, LRWC, and leaf area of the Na_2_CO_3_-stressed seedlings showed an appreciable decrease with the rise of Na_2_CO_3_ concentration. The maximum reduction in these growth parameters was recorded at the highest alkalinity level (75 mM Na_2_CO_3_) relative to untreated control plants (**Table 1**). Priming seeds with Si relieved the injurious impacts of alkaline stress on all the growth parameters examined, especially at the highest alkalinity level, at which the maximum recover was noted, as compared with stressed alone plants (**Table 1**). Pre-treatment of seeds with Si alone significantly increased the fresh weight, dry weight and leaf area; however, it did not significantly affect the LRWC compared with untreated control group (**Table 1**).

**Table 1:** Effects of different levels of alkaline stress with and without seed-priming with silicon (Si) on fresh weight (FW), dry weight (DW), leaf relative water content (LRWC), and leaf area of 25-day-old maize plants.

Treatments Na_2_CO_3_ (mM)	Si (1.5 mM)	FW (g plant^-1^)	DW (g plant^-1^)	LRWC (%)	Leaf area (cm^2^ plant^-1^)
0	– Si	1.45 ± 0.03^c^	0.24 ± 0.009^b^	83.67 ± 1.03^a^	25.38 ± 0.45^c^
	+ Si	1.85 ± 0.09^a^	0.28 ± 0.027^a^	84.54 ± 2.02^a^	30.30 ± 0.34^a^
25	– Si	1.28 ± 0.12^d^	0.22 ± 0.01^bc^	82.73 ± 2.00^ab^	23.22 ± 0.23^d^
	+ Si	1.68 ± 0.02^b^	0.27 ± 0.01^a^	84.17 ± 1.05^a^	28.54 ± 0.28^b^
50	– Si	0.76 ± 0.03^e^	0.15 ± 0.007^d^	79.73 ± 1.09^c^	18.37 ± 0.46^e^
	+ Si	1.29 ± 0.09^d^	0.21 ± 0.002^c^	83.39 ± 1.07^ab^	23.52 ± 0.45^d^
75	– Si	0.41 ± 0.01^f^	0.09 ± 0.004^e^	77.16 ± 1.03^d^	12.48 ± 0.38^f^
	+ Si	0.77 ± 0.03^e^	0.14 ± 0.009^d^	81.32 ± 1.06^bc^	18.42 ± 0.43^e^

*Values are means ±*standard deviation (SD)* of three independent replications (*n* = 3). Different letters within the column indicate statistically significant differences between treatments, according to Duncan’s multiple range test (*p* < 0.05)*.

### Priming with Si Improves the Contents of Photosynthetic Pigments

**FIGURE 1:**
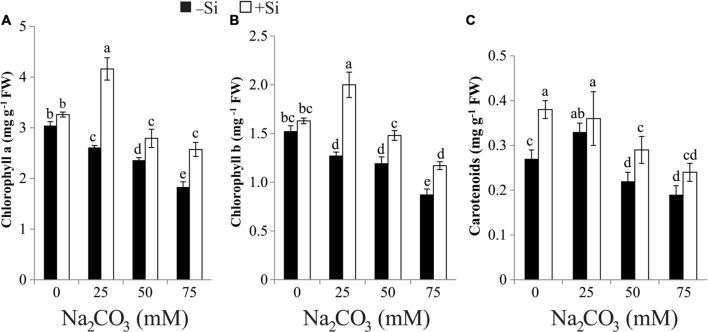
**Effects of different levels of alkaline stress with and without seed-priming with silicon (Si) on the contents of **(A)** chlorophyll a, **(B)** chlorophyll b and **(C)** carotenoids in leaves of 25-day-old maize plants**. Bars represent standard deviation (SD) of the means (*n* = 3). Different letters indicate significant differences among the treatments at *P* < 0.05, according to Duncan’s multiple range test. FW, fresh weight.

Results presented in **Figure 1** indicated that the increase in Na_2_CO_3_ concentration in the soil was associated with progressive fall in photosynthetic pigment biosynthesis in maize leaves relative to untreated control. On the other hand, the lowest level of Na_2_CO_3_ (25 mM) increased the biosynthesis of carotenoids compared with the untreated control. Priming maize seeds with Si amended the inhibitory effects of alkaline stress on pigment contents, resulting in significant enhancement of pigment contents in comparison with stressed alone plants (**Figure 1**). Under non-stressed regimes, the levels of Chl a and Chl b remained relatively unchanged but that of carotenoids increased by 40.74% in plants treated with Si alone versus untreated control (**Figure 1**).

### Si Increases the Levels of Soluble Sugars, Soluble Proteins, Total Phenols, and Total Free Amino Acids, While Restrains Proline Accumulation

The effects of alkaline stress on the contents of osmoprotectants and antioxidants varied. Soluble sugar content in maize plants was not significantly affected by Na_2_CO_3_ treatment at 25 mM, while it significantly decreased at 50 and 75 mM Na_2_CO_3_, compared with untreated control (**Table 2**). Although both soluble protein and total phenol contents remained unchanged up to 50 mM of Na_2_CO_3_, soluble protein content increased at 75 mM Na_2_CO_3_, whereas total phenol content progressively reduced at 75 mM Na_2_CO_3_ in comparison with untreated control (**Table 2**). Total free amino acid content slightly increased at 25 mM of Na_2_CO_3_, thereafter a constant increment in this content was recorded at 50 and 75 mM Na_2_CO_3_ compared with untreated control (**Table 2**). Alkaline stress induced a dramatic accumulation of proline, and this accumulation reached the maximum value at 75 mM Na_2_CO_3_ versus untreated control (**Table 2**).

**Table 2:** Effects of different levels of alkaline stress with and without seed-priming with silicon (Si) on the contents of soluble sugars, soluble proteins, total free amino acids, proline, and total phenols in 25-day-old maize plants.

Treatments Na_2_CO_3_ (mM)	Si (1.5 mM)	Soluble sugars (mg g^-1^ DW)	Soluble proteins (mg g^-1^ DW)	Total free amino acids (mg g^-1^ DW)	Proline (mg g^-1^ DW)	Total phenols (mg GAE g^-1^ DW)
0	– Si	25.87 ± 1.18^bc^	22.00 ± 2.14^c^	9.98 ± 0.53^e^	1.26 ± 0.08^e^	31.63 ± 1.02^c^
	+ Si	27.97 ± 1.02^b^	22.22 ± 0.47^c^	10.05 ± 0.06^e^	1.06 ± 0.05^f^	36.99 ± 0.95^b^
25	– Si	24.35 ± 1.81^c^	22.99 ± 2.46^c^	10.48 ± 0.38^e^	1.78 ± 0.13^c^	30.64 ± 0.93^c^
	+ Si	26.40 ± 1.00^bc^	25.69 ± 1.56^b^	15.01 ± 0.41^b^	1.16 ± 0.06^ef^	40.00 ± 1.80^a^
50	– Si	21.23 ± 0.89^d^	22.78 ± 0.52^c^	11.13 ± 0.23^d^	2.15 ± 0.09^b^	29.59 ± 0.36^c^
	+ Si	24.29 ± 1.32^c^	28.15 ± 0.87^b^	14.24 ± 0.13^c^	1.42 ± 0.06^d^	39.81 ± 2.33^a^
75	– Si	18.78 ± 1.31^e^	26.76 ± 0.88^b^	11.67 ± 0.33^d^	2.69 ± 0.04^a^	22.18 ± 0.33^d^
	+ Si	30.60 ± 1.12^a^	32.12 ± 1.09^a^	16.00 ± 0.28^a^	1.45 ± 0.03^d^	29.66 ± 0.16^c^

*Values are means ±*standard deviation (SD)* of three independent replications (*n* = 3). Different letters within the column indicate statistically significant differences between treatments, according to Duncan’s multiple range test (*p* < 0.05). DW, dry weight; GAE, gallic acid equivalent*.

Application of Si alleviated the adverse effects of alkaline stress on soluble sugar accumulation, especially at 75 mM Na_2_CO_3_ compared with stressed alone plants (**Table 2**). Supplementation with Si as seed-priming provoked the accumulation of soluble proteins, total phenols, and total free amino acids. The maximum increase in soluble protein and total phenol contents was noted at 50 mM Na_2_CO_3_, while that of total free amino acid content was observed at 25 mM Na_2_CO_3_ in maize plants treated with both Na_2_CO_3_ and Si, when compared with the stressed alone plants (**Table 2**). In contrary, application of Si in terms of seed-priming restrained the accumulation of proline in maize plants treated with both Na_2_CO_3_ and Si as compared with respective plants treated with Na_2_CO_3_ alone (**Table 2**). It is worth mentioning that under non-stressed conditions, only total phenol content was significantly affected by Si treatment, with an increase of 16.94% in comparison with untreated control (**Table 2**).

### Si Attenuates Na^+^ Toxicity and Modulates K^+^ Homeostasis and K^+^/Na^+^ Ratio

**FIGURE 2:**
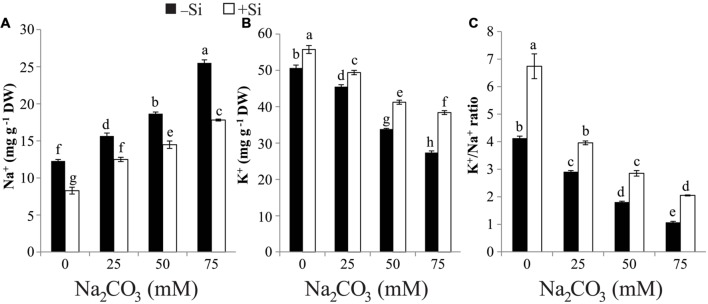
**Effects of different levels of alkaline stress with and without seed-priming with silicon (Si) on **(A)** Na^+^ content, **(B)** K^+^ content and **(C)** K^+^/Na^+^ ratio in 25-day-old maize plants**. Bars represent standard deviation (SD) of the means (*n* = 3). Different letters indicate significant differences among the treatments at *P* < 0.05, according to Duncan’s multiple range test. DW, dry weight.

The concentration of Na^+^ positively correlated with the increase of alkaline salt and recorded its highest value (twofold) at the severest alkalinity compared with control (**Figure 2A**). On the other hand, the reverse trend was noticed with K^+^ content and the highest reduction in K^+^ content was recorded at the highest level of Na_2_CO_3_ relative to that of control (**Figure 2B**). As a consequence, K^+^/Na^+^ ratio was reduced by increasing the concentration of Na_2_CO_3_ (**Figure 2C**). Si supplementation in the form of seed-priming resulted in significant repression in the accumulation of Na^+^ at all Na_2_CO_3_ concentrations. Furthermore, Si application increased the content of K^+^ and K^+^/Na^+^ ratio in stressed maize plants, with the maximum increase being observed at 75 mM Na_2_CO_3_ compared with the corresponding stressed alone plants (**Figures 2A–C**).

### Si Mitigates Na_2_CO_3_-Induced Oxidative Damage

**FIGURE 3:**
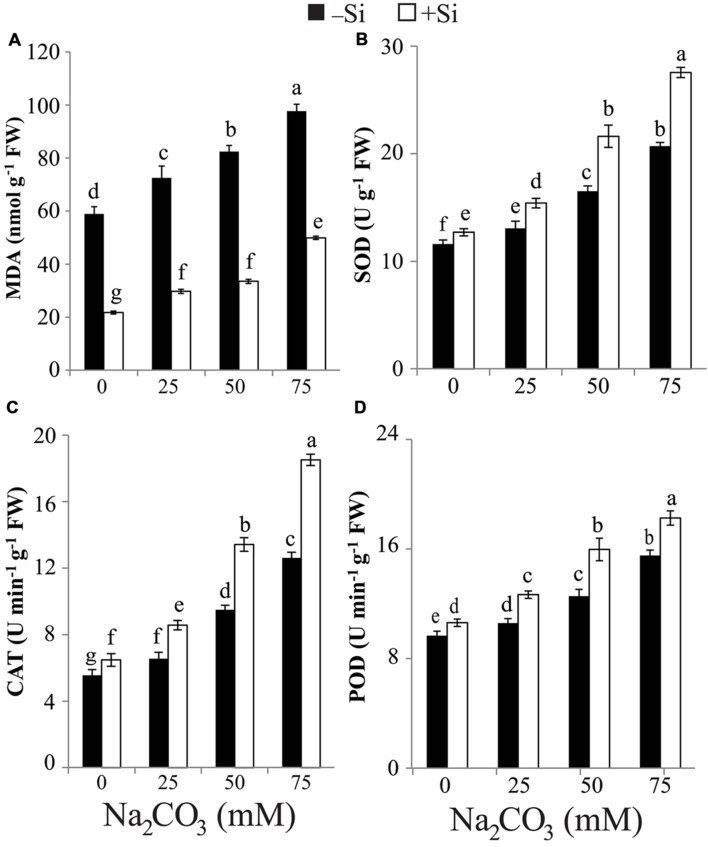
**Effects of different levels of alkaline stress with and without seed-priming with silicon (Si) on **(A)** the content of malondialdehyde (MDA) and the activities of **(B)** superoxide dismutase (SOD), **(C)** catalase (CAT) and **(D)** peroxidase (POD) in leaves of 25-day-old maize plants**. Bars represent standard deviation (SD) of the means (*n* = 3). Different letters indicate significant differences among the treatments at *P* < 0.05, according to Duncan’s multiple range test. FW, fresh weight.

The rise in alkalinity induced a significant accumulation in MDA content in stressed maize plants even at the lowest level of Na_2_CO_3_. The maximum accumulation in MDA content was attained at the highest level of alkalinity compared with untreated control plants (**Figure 3A**). Seed-priming with Si resulted in reduction in MDA content in Si-primed maize plants treated with any level of Na_2_CO_3_ relative to respective unprimed plants treated with Na_2_CO_3_ alone (**Figure 3A**).

### Si Increases the Activities of SOD, CAT, and POD to Mitigate Oxidative Stress

In comparison with control, the activities of SOD, CAT, and POD significantly increased in stressed maize plants with increasing levels of alkaline salt (**Figures 3B–D**). The highest increment in SOD, CAT, and POD activities was recorded in stressed alone maize plants at 75 mM Na_2_CO_3_ compared with untreated control plants. Si application induced further increase in the activities of SOD, CAT, and POD in Si-primed and stressed maize plants relative to stressed alone plants (**Figures 3B–D**). The maximum increase in the activities of SOD and CAT in Si + Na_2_CO_3_-treated plants was observed at 75 mM Na_2_CO_3_ (**Figures 3B,C**), while that of POD was recorded at 50 mM Na_2_CO_3_ (**Figure 3D**). Under non-stressed conditions, Si treatment also enhanced the activities of SOD, CAT, and POD in Si-treated plants versus untreated control (**Figures 3B–D**).

## Discussion

In this study, the impacts of Si as an agriculturally effective fertilizer element on mitigation of alkalinity pressure were studied in maize. The detrimental influences caused by alkaline salt on different growth parameters of maize plants could occur due to the raise in pH, reduction in cell enlargement and cell division, metabolic disorders, nutritional damage, and ion imbalance (**Table 1**) (Radi et al., 2012; Abd-Alla et al., 2014; Mohsenian and Roosta, 2015). The improvement of LRWC in stressed maize plants observed under Si treatment was perhaps due to the deposition of Si as silicate crystals in epidermal tissues, which composes a barrier to water transpiration through the cuticles and stomata (Habibi, 2015; Karmollachaab and Gharineh, 2015), resulting in higher leaf area of maize plants as recorded in Si-primed plants under non-stressed and alkaline stress conditions (**Table 1**). Furthermore, Si pre-treatment as seed-priming improved other growth parameters of alkaline-stressed maize seedlings (**Table 1**), strongly demonstrating the ameliorating action of Si in minimizing alkaline stress. Thus, the results of this study and previously published reports collectively indicate the protecting role of Si against a wide range of environmental pressures (Farooq et al., 2013; Habibi and Hajiboland, 2013; Tripathi et al., 2013; Shen et al., 2014).

The depression in photosynthetic pigments of maize leaves during Na_2_CO_3_ treatment found in this study (**Figure 1**) was supported by the findings of Radi et al. (2012). The decrease in Chl contents under alkaline stress might be due to (i) Mg^2+^ precipitation that results in the degradation of green pigments (Shi and Zhao, 1997), (ii) enhanced oxidative stress that causes injury to chloroplast structure, and (iii) increase in the activity of chlorophyllase enzyme that is responsible for the Chl degradation (Kariola et al., 2005; Abdel Latef and Chaoxing, 2011; Abdel Latef et al., 2014; Mostofa et al., 2015). Additionally, alkaline stress decreased the content of carotenoids in stressed maize plants (**Figure 1C**), which act as effective scavengers of free radicals provoked by reactive oxygen species (ROS; Gururani et al., 2015). It is worth noting that, the increase of carotenoid content observed by 25 mM Na_2_CO_3_ treatment in stressed maize plants could improve the amplitudes of this compound to diminish the damage caused by ROS under low level of alkaline salt. Si augmented the Chl and carotenoid contents in maize plants exposed to alkaline stress, which could be resulted from the increase in leaf area, leading to the increase in green pigments per unit area and safeguarding of Chl pigments from ROS by reinforcing the level of carotenoids (**Figure 1**; **Table 1**).

Importantly, Si-induced restoration of the levels of LRWC and photosynthetic pigments in the alkaline-stressed maize seedlings demonstrated an osmoprotective and membrane-protecting role of Si for maize seedlings subjected to alkaline stress (**Figure 1**; **Table 1**). The decrease in both soluble sugar and Chl contents under alkaline stress provides a reason to believe that low Chl content causes a relevant reduction of light absorption by leaves, and subsequently reduces the soluble sugar content (**Figure 1**; **Table 2**) (Hamdia et al., 2004; Hamdia and Shaddad, 2010). The amelioration in osmoregulation potential in terms of increasing soluble sugar content by Si could be highly associated with enhanced photosynthetic activity and better growth under alkaline conditions (**Figure 1**; **Tables 1** and **2**). This finding suggests that pre-treatment of maize seeds with Si might enhance the catabolism of starch to soluble sugars under alkaline stress.

The accumulation of soluble proteins in maize plants under high alkaline pressure may supply a storage form of nitrogen that is reutilized when stress is over (Abdel Latef, 2010; Abdel Latef and Chaoxing, 2014; Zhang et al., 2014). The increase in soluble protein content of maize under the highest alkaline level applied was accompanied by a marked reduction in growth (**Tables 1** and **2**). This finding suggests that under alkaline stress, maize plants divert most of the synthesized proteins from being used for growth to being used for osmoregulation to survive stress. It is noteworthy that the high soluble protein content observed at 75 mM Na_2_CO_3_ was not resulted from reduction of total free amino acid and proline contents (**Table 2**). Thus, the strategy that maize plants use to cope with alkaline stress could be the enhancement of nitrogen metabolism. The increase in soluble protein content in response to Si application may be due to the facts that (i) Si has a pivotal role in binding amino acids to form specific proteins (Soundararajan et al., 2014), and (ii) Si is actively engaged in formation of DNA and functioning of mRNA (Abbas et al., 2015). In addition, seed-priming with Si reduced the accumulation of proline content in the alkaline-stressed seedlings, which is associated with improved growth of maize plants under alkaline stress (**Tables 1** and **2**). This result suggested that seed-priming with Si could provide protection to cells by keeping the accumulation of proline to an optimum level, and Si probably employed other osmoprotectants for stress mitigation, in which high level of proline accumulation was not required.

In the present study, increase of alkaline stress provoked an increment in the Na^+^ content and a depression in the K^+^ content, resulting in reduction in K^+^/Na^+^ ratio (**Figures 2A–C**). The decrease in K^+^ uptake under alkaline stress may be due to the repressive influence of the stress on the absorption of this cation and competition of Na^+^ ion with K^+^ ion for binding sites needful for various cellular functions (Azooz et al., 2015). Si-primed maize plants significantly decreased Na^+^ concentration, while improved the K^+^ concentration, and thus increasing the K^+^/Na^+^ ratio (**Figure 2**). This reduction in Na^+^ accumulation might be due to the deposition of silicate in the exodermis and endodermis of maize roots, which leads to an obstruction of the apoplastic Na^+^ absorption by roots under alkaline stress (Wang et al., 2015). The possible reason for the increased K^+^ uptake could be the promotion effect of Si on the plasma membrane H^+^-ATPases in the roots, as found in wheat under water deficit (Karmollachaab and Gharineh, 2015). The high K^+^/Na^+^ ratio is a good sign on the balancing impacts of Si on K^+^ and Na^+^ uptakes under alkaline stress, encouraging the application of Si as seed-priming for plants grown on alkaline soils.

Under alkaline regimes, MDA content accumulated in maize plants (**Figure 3A**), clearly suggesting ROS burst and prospective oxidative damage to plant cells. This result is supported by the study of Ahmad et al. (2014), who found an increase in MDA content in two mulberry (*Morus alba* L.) cultivars with increase in exogenous NaHCO_3_ level. On the other hand, treatment with Si significantly hampered MDA accumulation in stressed maize plants compared with that of control and Na_2_CO_3_- treated alone plants (**Figure 3A**), suggesting that Si triggers mechanisms to mitigate oxidative damage in stressed plants. Indeed, Si pre-treatment significantly increased the content of antioxidant phenols in maize plants under alkaline stress (**Table 2**). Furthermore, seed-priming with Si also resulted in a significant increase in SOD, CAT, and POD activities in stressed maize plants relative to plants treated with Na_2_CO_3_ alone (**Figures 3B–D**). These results indicate that Si enhances antioxidant system to protect plants against alkalinity-induced oxidative damage, as evidenced by the observed reduced MDA level (**Figure 3A**).

**FIGURE 4:**
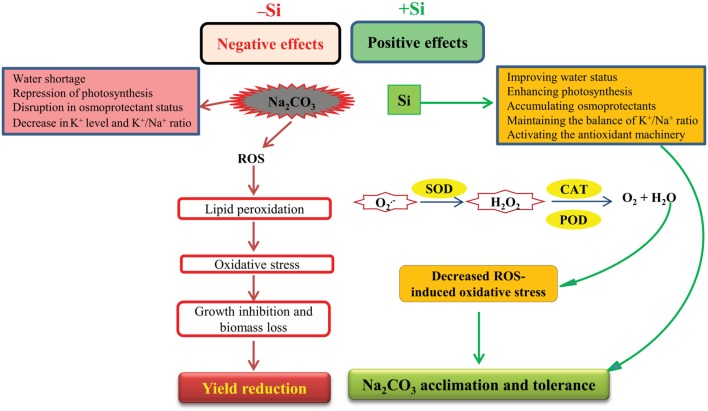
**A schematic diagram representing effects of alkaline stress (e.g., Na_2_CO_3_ stress) and defensive system induced by seed-priming with Si, underlying maize alkaline stress tolerance**. Alkaline stress causes negative impacts by repressing photosynthesis, disrupting osmoprotectant status and negatively influencing water status and ionic balance. Excessive alkalinity can promote production of reactive oxygen species (ROS). ROS contribute to lipid peroxidation, resulting in oxidative stress that causes growth inhibition, biomass loss, and subsequently extreme yield reduction. Conversely, seed-priming with Si shows protective mechanism against alkaline stress by improving water balance and photosynthesis, accumulating osmoprotectants and maintaining ionic balance. Si also enhances the detoxification of ROS by stimulating the activities of antioxidant enzymes, such as SOD, CAT, and POD, leading to oxidative stress mitigation. As a result, Si improves alkaline stress tolerance by retaining better growth of alkaline-stressed plants. $O_{2}^{•-}$, superoxide; H_2_O_2_, hydrogen peroxide.

In view of all the above observations, a schematic diagram was delineated in **Figure 4**, which represents the possible physiological and biochemical strategies involving alkalinity-induced stress and the Si-mediated alkaline stress tolerance in maize plants. To our knowledge, this is the first report dealing with the effects of Si on growth parameters, antioxidant activities and selected physiological and biochemical characteristics of maize plants under alkaline stress. Our results demonstrate the potential application of Si in dealing with alkaline stress. Further efforts are required to gain a full understanding of how Si amends the alkaline stress responses in plants.

## Author Contributions

AA conceived, designed, conducted the experiments, collected and analyzed the data. AA and L-ST wrote and revised the manuscript.

## Conflict of Interest Statement

The authors declare that the research was conducted in the absence of any commercial or financial relationships that could be construed as a potential conflict of interest.
